# Tear proteomic analysis in keratoconus patients and potential biomarkers: a case-control study

**DOI:** 10.3389/ebm.2025.10864

**Published:** 2026-01-12

**Authors:** Daniel de Almeida Borges, Marcos Rodrigo Alborghetti, Romenia Ramos Domingues, Adriana Franco Paes Leme, Mônica Alves

**Affiliations:** 1 Department of Ophthalmology and Otorhinolaryngology, Faculty of Medical Sciences, University of Campinas (UNICAMP), Campinas, São Paulo, Brazil; 2 Brazilian Biosciences National Laboratory (LNBio), Brazilian Center for Research in Energy and Materials (CNPEM), Campinas, Brazil

**Keywords:** biomarkers, cornea, keratoconus, proteomics, tear film

## Abstract

Keratoconus is a corneal ectasia whose pathophysiological mechanisms, including biomolecular alterations and genetic influences, remain poorly understood. Recent studies have shown altered cytokine levels, increased proteinase activity, and other potential mediators in the tear film and corneal tissue, highlighting a possible involvement of inflammatory pathways in the pathophysiology of keratoconus. This observational study aims to characterize the tear proteome of keratoconus patients and compare it to a control group, reporting potential disease biomarkers in the tear film. 23 keratoconus patients were selected at the Cornea and External Diseases Outpatient Clinic of the Clinics Hospital of UNICAMP. The control group consisted of 17 age- and sex-matched participants. All study subjects underwent corneal tomography (Pentacam). Tear film samples were collected and sent for proteomic evaluation by mass spectrometry at the National Biosciences Laboratory (LNBio). After quantification, univariate and multivariate statistical analyses were performed. A total of 353 proteins were identified and quantified, of which 25 showed statistical differences in the univariate analysis (t-test), and 19 were selected in the multivariate analysis (PLS-DA). There was an overlap of 7 proteins identified in both uni- and multivariate analyses: chitinase-3-like protein 2, prosaposin, zymogen granule protein 16 homolog B, procollagen-lysine,2-oxoglutarate 5-dioxygenase 1, secretoglobin family 1D member 1, albumin, and Ig kappa chain V-I region. Thirty-seven proteins showed statistically significant variation between the keratoconus and control groups. Proteomic analysis revealed differentially expressed proteins in the tear film of keratoconus patients. We report the identified proteomic profile, which includes potential biomarkers that may help elucidate the disease’s pathophysiology.

## Impact statement

This study provides a comprehensive proteomic characterization of the tear film in keratoconus, identifying differentially expressed proteins that reveal novel insights into the disease’s molecular mechanisms. By combining univariate and multivariate statistical analyses, including PLS-DA, we demonstrated a distinct proteomic signature that discriminates keratoconus from controls, implicating inflammatory, oxidative stress, and extracellular matrix remodeling pathways. These findings advance the field by expanding the catalog of candidate biomarkers and highlighting molecular pathways that may contribute to disease onset and progression. The data support the concept that keratoconus is not merely a structural ectasia but a complex disorder involving active biochemical dysregulation. This work provides a valuable proteomic resource for the ophthalmic research community and lays the groundwork for future translational studies aimed at early diagnosis and the development of targeted therapeutic strategies.

## Introduction

Keratoconus is a corneal ectasia defined by progressive corneal thinning and protrusion, leading to irregular astigmatism and varying degrees of visual impairment. It typically manifests during puberty and progresses until the third or fourth decade of life. It is usually bilateral, often asymmetrical, though rarely presented unilaterally [1–3]. Keratoconus remains one of the leading indications for corneal transplantation worldwide [2] and has a significant financial impact as it affects economically active individuals [4].

A recent meta-analysis, which included 29 studies and a total population of over 50 million individuals from 15 different countries, found a global prevalence of 1.38 cases per 1,000 inhabitants [4]. Prevalence rates vary widely, reflecting differences in sample sizes, diagnostic methodologies, and the influences of genetic and ethnic factors in keratoconus presentation [4]. No population-based studies have been found on the prevalence of keratoconus in Brazil.

The main risk factors associated with keratoconus include eye rubbing, allergy, asthma, eczema, and a positive family history [3, 4], highlighting genetic and environmental influences. Approximately 8%–10% of keratoconus cases have a positive family history [2, 3]. Substantial evidence supports a genetic influence in keratoconus pathogenesis [2, 5, 6]. Twin studies provide insights into the relative contributions of genotype and environment to disease phenotypic expression. Keratoconus in monozygotic twins demonstrates high concordance with variable expressivity [1, 7, 8]. Studies in families of keratoconus patients have revealed topographic and tomographic abnormalities even in asymptomatic individuals [9, 10]. Indeed, a first-degree relative with keratoconus is considered a major risk factor for disease development [10]. Recent studies have identified over 20 genes implicated in keratoconus susceptibility [11]. Keratoconus is currently regarded as a multifactorial disease, where multiple genetic factors interact with environmental influences to determine its clinical presentation [12]. Recent proteomic work in offspring of patients with keratoconus further supports this concept, demonstrating early molecular alterations, particularly involving oxidative stress responses, cytoskeletal organization, and mechanotransduction, even before clinical or biomechanical abnormalities become detectable [13].

Keratoconus is a disease with a significant socio-economic impact due to its relatively high prevalence and its effect on an economically active age group. Despite being described nearly 300 years ago, the detailed pathophysiological, genetic, and environmental mechanisms involved in its onset and progression remain unclear. Current treatment is based on visual rehabilitation, ranging from optical aids such as glasses and rigid or scleral contact lenses to surgical interventions, including intrastromal rings and corneal transplantation [3]. Corneal crosslinking aims to halt disease progression by altering the structural properties of stromal collagen. The only preventive approach is controlling known risk factors, such as eye rubbing and ocular allergy. No pharmacological treatment is available to interfere with the altered metabolic pathways of the disease, nor is there a susceptibility test to identify individuals at risk of developing keratoconus or those already diagnosed at risk of progression [3].

Although keratoconus has been initially described as a noninflammatory disease due to the absence of clinical signs of inflammation, such as conjunctival hyperemia, corneal infiltrates, or anterior chamber reaction [1, 14], later studies have reported significant alterations in inflammatory mediators, including increased levels of pro-inflammatory cytokines, collagen degradation enzymes such as matrix metalloproteinases, indicating that keratoconic corneas exhibit some degree of inflammation [12, 15–18]. Oxidative stress markers and antioxidant systems are also dysregulated in keratoconus. Evidence suggests an increase in oxidative stress markers, particularly reactive oxygen species (ROS) and reactive nitrogen species (RNS), alongside a reduction in antioxidants such as ALDH/NADPH dehydrogenase, lactoferrin, transferrin, albumin, selenium, zinc, vitamin B12, and folic acid, among others [19, 20]. Large-scale proteomic studies have reinforced this inflammatory and oxidative-stress profile by identifying dysregulation of tear proteins involved in glycolytic pathways, reactive oxygen detoxification, and inflammatory regulation across different disease stages, including cystatin-S, lacritin, glutathione synthetase, and superoxide dismutase [21].

Proteomic analysis of human tissues and fluids has emerged as one of the most relevant recent approaches in biomarker research. The tear film has garnered increasing interest in recent years as a potential source of biomarkers for various diseases due to its accessibility, moderate complexity, and responsiveness to both ocular and systemic diseases [22]. Searching for biomarkers with high sensitivity and specificity for a given disease is crucial to improving diagnostic methods, identifying cellular and metabolic alterations that may elucidate underlying pathophysiological mechanisms, and providing potential therapeutic targets [23].

There is growing evidence that keratoconus exhibits a characteristic proteome [24]. Collagen and other structural proteins, such as lumican, keratan sulfate, and decorin, are decreased [24]. Conversely, there is an increased expression of degradative enzymes, including phosphatases, lipases, esterases, cathepsins, and matrix metalloproteinases, as well as elevated levels of pro-inflammatory proteins such as interleukin 1 (IL-1), interleukin 6 (IL-6), matrix metalloproteinase 9 (MMP-9), transforming growth factor beta (TGF-ß), and tumor necrosis factor alpha (TNF-α) [16, 17, 25]. Complementary evidence indicates that impaired epithelial wound healing, dysregulated epithelial-mesenchymal transition pathways, and altered cytokine signaling contribute to the characteristic topographic changes and epithelial remodeling observed in keratoconus [26].

In a previous study [27], we demonstrated that mass spectrometry-based proteomics performed on tear samples was able to differentiate three distinct diseases: keratoconus, pterygium, and dry eye secondary to graft-versus-host disease (GVHD). Each disease exhibited a characteristic proteomic profile, identifiable through multivariate statistical analysis methods. Furthermore, we identified the main differentially expressed proteins in each group compared to the control, which were reported as potential biomarkers for each disease. In our previous evaluation, the keratoconus group consisted of four samples, and the control group included six samples, which have been expanded in the present study.

Despite recent discoveries, the exact mechanisms initiating the cellular and molecular alterations that culminate in corneal degradation and shape distortion in keratoconus remain unknown. Furthermore, the interactions between genetic and environmental factors in modulating these alterations are yet to be fully elucidated. Tear proteomic analysis can be an important tool in the search for biomarkers in ocular diseases and has been used to investigate the pathophysiology of keratoconus.

This study aims to quantify and report differentially expressed proteins in the tear film of patients with keratoconus compared to a control group.

## Materials and methods

This is a cross-sectional, observational, and non-interventional study. The research subjects were divided into two groups: 23 patients diagnosed with keratoconus under follow-up at the Cornea and External Diseases Outpatient Clinic of the Clinics Hospital of UNICAMP, and 17 control subjects. All research subjects underwent a comprehensive ophthalmological examination and corneal tomography using the OCULUS Pentacam® software 1.20r134. The diagnosis in the Keratoconus group was confirmed by identifying characteristic signs, including increased corneal curvature, stromal thinning, alterations in elevation maps, and irregular astigmatism. Corneal tomography was also used in the Control group to confirm the absence of the disease.

Patients diagnosed with keratoconus and under follow-up at the Keratoconus Outpatient Clinic of HC/UNICAMP who consented to participate in the study were included. For the control group, volunteers were recruited from hospital personnel, university students, and family members of patients from other ambulatories, all without clinical or tomographic signs of keratoconus, without a family history of the disease, and without any ocular surface pathology. Subjects were excluded if they were using anti-inflammatory, immunosuppressive, or immunomodulatory medications, either topical or systemic, or had active ocular inflammatory or infectious conditions at the time of data collection. Additionally, individuals with a history of ocular surface surgery, including laser refractive surgery, radial keratotomy, intrastromal ring implantation, corneal crosslinking, pterygium excision, cataract surgery, among others, were excluded, as well as previous trauma or signs of traumatic corneal scarring.

Tear samples were collected using microcapillary pipettes with atraumatic contact with the lower tear meniscus. The samples were then transferred to cryotubes and frozen at −80 °C. The samples (17 controls and 23 keratoconus cases) were later prepared and processed as previously described [27, 28].

Following data acquisition, processing was performed using MaxQuant software version 1.3.0.3 with the Andromeda algorithm against the UniProt Human Protein Database (downloaded in May 2019, containing 95,542 sequences and 38,078,700 residues). Bioinformatics analyses were conducted using Perseus software version 1.5.1.6. Logarithmic transformation was applied, and filters removed reverse sequences and proteins identified by only one modified peptide.

Mass spectrometry data underwent logarithmic transformation before statistical analysis. Univariate analyses were conducted using GraphPad Prism version 6.00. Measurements from keratoconus patient samples were compared with those of control samples using an unpaired t-test, both with and without correction for multiple analyses: false discovery rate 5% (FDR). Multivariate analyses were performed using the online platform MetaboAnalyst^1^ [29].

## Results

A total of 40 individuals were evaluated, divided into a keratoconus patient group (n = 23) and a control group (n = 17). The main clinical characteristics of each group are shown in Table 1.

**TABLE 1:** Clinical characteristics.

Variables	Keratoconus	Control	P-value
Sample size	23	17	​
Sex (female/Male)	10/13	11/6	0.184
Age (years)	21.65	24.47	0.075
Kmax (diopters)	57.7D	44.9D	<0.001
Pachymetry (µm)	454	566	<0.001
Belin D (pentacam)	9.56	0.94	<0.001
Corneal astigmatism (diopters)	4.51	1.16	<0.001
Ocular allergy	95.6%	23.5%	<0.001

**FIGURE 1:**
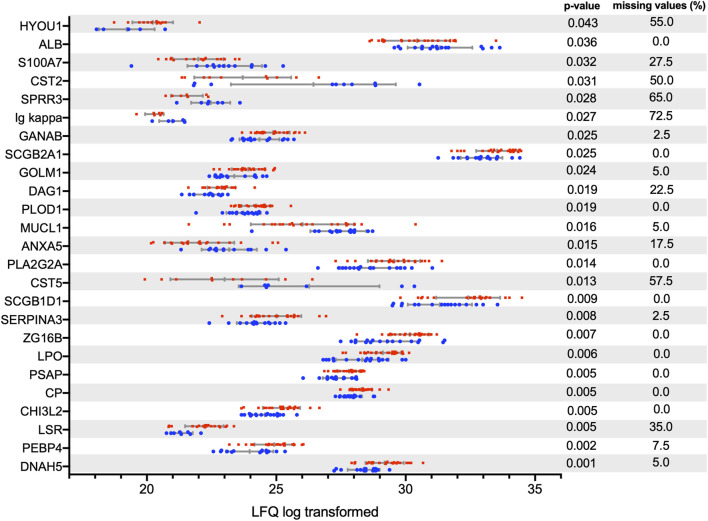
Tear proteins (identified by gene name) with a p-value <0.05 after Student's t-test without FDR correction. After FDR correction, no protein reached p-value <0.05. Data distribution with standard deviation and mean are displayed. Blue: control. Red: keratoconus patients.

**FIGURE 2:**
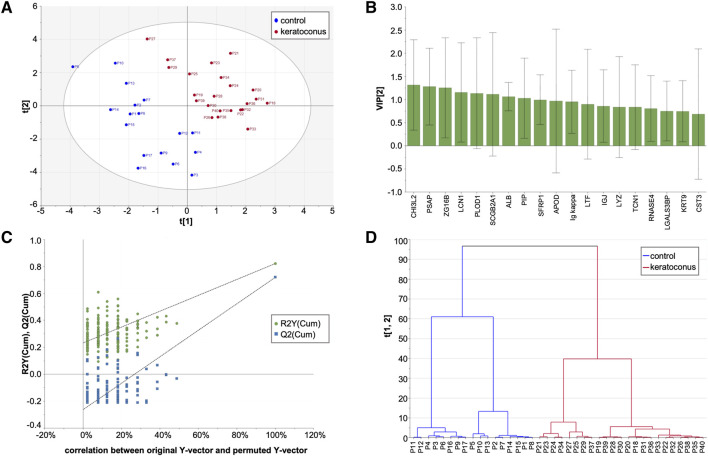
PLS-DA and hierarchical clustering analysis. No data imputation was performed, and variables (proteins) with missing values were excluded. **(A)** PLS-DA score plots of keratoconus and control groups: 2 latent variables, R2X = 0.433, R2Y = 0.822, Q2 = 0.720. **(B)** Variable influence on this classification (VIP–variable importance in the prediction). **(C)** Permutation test of PLS-DA models, intercepts: R2=(0.0, 0.232), Q2=(0.0, −0.263). **(D)** Hierarchical clustering analysis based on the variables selected in the PLS-DA test, shown in Panel **B**.

A total of 353 proteins were identified and quantified, of which 25 showed statistically significant differences in the univariate analysis using the t-test with p < 0.05 (Table 2; Figure 1), and 19 were selected in the multivariate partial least-squares discriminant analysis (PLS-DA) (Figure 2). Seven proteins were identified in both the uni- and multivariate analyses: chitinase-3-like protein 2, prosaposin, zymogen granule protein 16 homolog B, procollagen-lysine,2-oxoglutarate 5-dioxygenase 1, secretoglobin family 1D member 1, albumin, and Ig kappa chain V-I region. Thirty-seven proteins exhibited statistically significant variation between the keratoconus and control groups.

**TABLE 2:** Differentially expressed proteins after t-test with P < 0.05.

Gene	UniProt ID	Protein name	P-value	Control/KC ratio
DNAH5	Q8TE73	Dynein heavy chain 5, axonemal	0.0011	0.54
PEBP4	Q96S96	Phosphatidylethanolamine-binding protein 4	0.0017	0.55
LSR	Q86X29	Lipolysis-stimulated lipoprotein receptor	0.0047	0.50
CHI3L2	Q15782	Chitinase-3-like protein 2	0.0049	0.62
CP	P00450	Ceruloplasmin	0.005	0.76
PSAP	P07602	Prosaposin	0.005	0.74
LPO	P22079	Lactoperoxidase	0.006	0.65
ZG16B	Q96DA0	Zymogen granule protein 16 homolog B	0.0069	0.69
SERPINA3	P01011	Alpha-1-antichymotrypsin	0.0083	0.52
SCGB1D1	O95968	Secretoglobin family 1D member 1	0.0088	0.49
CST5	P28325	Cystatin-D	0.0134	18.04
PLA2G2A	P14555	Phospholipase A2, membrane-associated	0.014	0.60
ANXA5	P08758	Annexin A5	0.0145	1.77
MUCL1	Q96DR8	Mucin-like protein 1	0.0164	1.31
PLOD1	Q02809	Procollagen-lysine,2-oxoglutarate 5-dioxygenase 1	0.0187	0.73
DAG1	Q14118	Dystroglycan; Alpha-dystroglycan; Beta-dystroglycan	0.0192	0.70
GOLM1	Q8NBJ4	Golgi membrane protein 1	0.0237	0.72
SCGB2A1	O75556	Mammaglobin-B	0.0253	0.67
GANAB	Q14697	Neutral alpha-glucosidase AB	0.0253	0.72
Ig kappa	P01597	Ig kappa chain V-I region	0.0265	1.65
SPRR3	Q9UBC9	Small proline-rich protein 3	0.0284	1.95
CST2	P09228	Cystatin-SA	0.031	12.11
S100A7	P31151	Protein S100-A7	0.032	2.38
ALB	P02768	Serum albumin	0.0359	1.79
HYOU1	Q9Y4L1	Hypoxia up-regulated protein 1	0.0433	0.54

## Discussion

The present study evaluated the tear proteome of patients with keratoconus compared to a control group without the disease using mass spectrometry and reported differentially expressed proteins between the groups.

Proteomics studies have been used to identify biological markers across various medical fields, including ophthalmology. In 2020, our group published a study [27] on proteomic analysis of tears from patients with keratoconus, pterygium, and dry eye associated with GVHD, in which we reported the tear proteome for each disease and the differentially expressed proteins in each group compared to controls. Multivariate statistical analyses of tear proteome distinguished each distinct ocular disease by a characterizable proteomic profile. Recently, a meta-analysis of candidate proteins associated with keratoconus [27] analyzed 346 normal and 493 keratoconus eyes. Altered proteins involved in inflammatory pathways, extracellular matrix remodeling, and apoptosis were reported. The main proteins identified were MMP-9, IL-6, lysyl oxidase (LOX), TNF, and IL-1B.

Below is a discussion of the study’s main findings, based on the UniProt online database^2^ and literature review. Table 3 summarizes the biological functions of the main proteins.

**TABLE 3:** Main biological functions of differentially expressed proteins identified in the tear film of keratoconus patients.

Gene	Protein name	Main biological function
PEBP4	Phosphatidylethanolamine-binding protein 4	Modulates PI3K-AKT signaling; involved in cell survival
CHI3L2	Chitinase-3-like protein 2	Binds to carbohydrate structures, may play a role in immune response and tissue remodeling
CP	Ceruloplasmin	Ferroxidase activity; antioxidant defense; iron and copper metabolism
PSAP	Prosaposin	Lysosomal degradation of sphingolipids; neurotrophic activity
LPO	Lactoperoxidase	Antimicrobial defense; oxidative stress marker
ZG16B	Zymogen granule protein 16 homolog B	Secreted protein; proposed role in sustaining pro-inflammatory environments
SERPINA3	Alpha-1-antichymotrypsin	Serine protease inhibitor; anti-inflammatory, antifibrotic, antioxidant roles
SCGB1D1	Secretoglobin family 1D member 1 (Lipophilin A)	Modulates inflammation and tissue repair
SCGB2A1	Mammaglobin B (Lipophilin C)	Secretory protein; androgen-binding; component of tear film protein complexes
CST5	Cystatin D	Inhibitor of cysteine proteases; protects extracellular matrix from degradation
CST2	Cystatin SA	Regulates proteolytic activity; contributes to tissue homeostasis
CST3	Cystatin C	Key inhibitor of cysteine proteases; modulates extracellular proteolysis
PLA2G2A	Secretory phospholipase A2	Hydrolyzes phospholipids; initiates arachidonic acid-mediated inflammatory pathways
ANXA5	Annexin A5	Epithelial repair; ECM structural organization
MUCL1	Mucin-like 1	Tear film lubrication and ocular surface protection
PLOD1	Procollagen-lysine,2-oxoglutarate 5-dioxygenase 1	Collagen fiber assembly and crosslinking
DAG1	Dystroglycan 1	Basement membrane organization; epithelial adhesion and signaling
SPRR3	Small proline-rich protein 3	Terminal epithelial differentiation; structural support
ALB	Albumin	Metal transport; antioxidant buffering
S100A7	Protein S100-A7 (psoriasin)	Inflammation, angiogenesis, oxidative stress regulation
HYOU1	Hypoxia up-regulated protein 1	Endoplasmic reticulum stress response; cytoprotection under hypoxia/inflammation

The proteomic profile in the tear film of keratoconus patients shows dysregulation of proteins involved in inflammation, oxidative stress, tissue remodeling, and extracellular matrix homeostasis. These mechanisms are widely implicated in the disease’s pathophysiology. Rather than evaluating each differentially expressed protein in isolation, a pattern emerges in which multiple proteins converge on interconnected biological processes that may underlie the corneal thinning, epithelial instability, and stromal degradation seen in keratoconus.

Several upregulated proteins, including ceruloplasmin, lactoperoxidase, and prosaposin, suggest an enhanced inflammatory and oxidative environment on the ocular surface. Increased oxidative stress markers have been consistently reported in keratoconus corneas [19, 30]. Our findings reinforce this biochemical signature. The upregulation of enzymes such as phospholipase A2 further suggests a heightened pro-inflammatory state [31, 32], consistent with studies reporting increased levels of inflammatory mediators in dry eye disease and in subsets of keratoconus patients with allergic comorbidities. Albumin, however, showed decreased levels in the keratoconus group, consistent with previous studies [19, 33–35]. Its reduced presence in tears may increase susceptibility to oxidative stress [19]. S100 A7 protein is linked to ocular surface inflammation [36] and recurrent pterygium [37], but its levels were lower in the keratoconus group.

Proteins associated with tissue remodeling also showed dysregulation; higher levels of procollagen-lysine,2-oxoglutarate 5-dioxygenase 1, and dystroglycan 1 suggest altered extracellular matrix turnover and basement membrane dynamics [38], processes already implicated in epithelial fragility and stromal biomechanical weakening in keratoconus. Proteins with potential roles in maintaining epithelial integrity, such as Annexin A5 [39, 40], mucin-like protein 1 [41], and small proline-rich protein 3 [42], were reduced in keratoconus tears, consistent with previous reports of compromised epithelial barrier function in these patients. Cystatins (CST2, CST3, CST5), key inhibitors of cysteine proteases, were also decreased, potentially contributing to increased proteolytic activity and stromal degradation—an established pathogenic mechanism in keratoconus [43–45]. SerpinA3, which has been demonstrated to exhibit anti-inflammatory, anti-angiogenic, antioxidant, and anti-fibrotic activities [46], was upregulated in the keratoconus group, perhaps as a response to the proinflammatory environment. The behavior of secretoglobins [47], however, differed from prior studies that reported reduced levels [43, 48]; their elevation in our cohort may reflect heterogeneity among patient populations or differences in disease stage, severity, or environmental exposures. Secretoglobin 2A1 has previously been reported to be upregulated in patients with keratoconus [49].

Some proteins associated with known risk factors for keratoconus were upregulated in the disease group. Hypoxia up-regulated protein 1, which is correlated with vernal keratoconjunctivitis [50], may be associated with the high prevalence of ocular allergy in this population. Saposins are associated with the sphingolipid metabolic pathway, which has been linked to eye-rubbing behavior [51].

Some proteins, such as chitinase-3-like protein 2 and zymogen granule protein 16 homolog B, remain poorly characterized in ocular physiology [49, 52] but were consistently upregulated in our analysis. Their repeated identification across proteomic studies suggests they may represent underexplored components of the inflammatory and remodeling responses in keratoconus.

Together, these differentially expressed proteins support a multifactorial disease model in which chronic inflammation, oxidative imbalance, epithelial barrier disruption, and extracellular matrix instability act synergistically to promote corneal thinning. Continued investigation into these pathways may help refine biomarkers for diagnosis and progression monitoring, as well as identify new therapeutic targets.

The PLS-DA model constructed to discriminate between keratoconus and control tear proteomes demonstrated strong explanatory and predictive performance. Using two latent variables, the model achieved R^2^X = 0.433, R^2^Y = 0.822, and Q^2^ = 0.720. The proportion of explained variance in the predictor matrix (R^2^X) indicates that the latent variables efficiently captured the most relevant structure within the proteomic dataset. The high R^2^Y value demonstrates that the model accounts for the majority of variance associated with group separation, supporting that the discrimination observed is largely driven by biological differences rather than random noise. Moreover, the Q^2^ value of 0.720 confirms the model’s high predictive capacity, suggesting that it can reliably classify new samples according to disease status.

Model validity was further supported by permutation testing, which yielded intercepts of R^2^ = 0.232 and Q^2^ = −0.263. The low R^2^ and negative Q^2^ intercepts from randomized models indicate that the original model’s performance was not due to overfitting, but rather reflects genuine structure–response relationships within the data. Together, these findings provide strong evidence that the proteomic profiles of keratoconus and control groups are distinctly segregated in the multivariate space, underscoring the robustness and biological relevance of the discriminatory model.

The main limitation of this study is that the findings from the univariate analysis did not remain statistically significant after FDR correction. This is due to the small sample size, which reduces the study’s power. However, the literature shows that proteomics studies using mass spectrometry rarely have larger sample sizes due to such analyses’ high cost and time-consuming nature. It is also important to make the current findings available, including raw proteomics data, as these can be used in meta-analyses and bioinformatics tools to contribute to a larger database.

Another limitation is that no filtering of differentially expressed proteins was performed based on the percentage of missing values among subjects or fold-change thresholds.

Our study demonstrated altered levels of several proteins related to inflammatory pathways and oxidative stress, which agrees with recent findings in the literature. Building on our previous study, we conducted a second proteomic evaluation of tear samples from keratoconus patients, with a sample size four times larger, providing further data for future biomarker analyses and aiding the scientific community in unraveling the intricate pathophysiological mechanisms involved in keratoconus. This study represents a significant step forward in elucidating the complex pathophysiological mechanisms underlying keratoconus. Identifying differentially expressed proteins in the tear film might serve as insight for potential biomarkers. By expanding the understanding of molecular alterations associated with the disease, our findings reinforce the role of inflammation and oxidative stress in keratoconus progression and pave the way for future translational research aimed at improving early diagnosis, risk stratification, and the development of targeted therapeutic interventions. Given the socio-economic burden of keratoconus and the current lack of disease-modifying treatments, identifying specific proteomic signatures in tear fluid holds immense potential for transforming clinical management in corneal ectatic diseases.

## Conclusion

Proteomic analysis revealed differentially expressed proteins in the tear film of keratoconus patients. We report the identified proteomic profile, which includes potential biomarkers that may help elucidate the disease’s pathophysiology, providing data for future studies.

## Data Availability

The datasets presented in this study can be found in online repositories. The names of the repository/repositories and accession number(s) can be found in the article/Supplementary Material.

## Footnotes

1
https://www.metaboanalyst.ca

2
https://www.uniprot.org
